# Comprehensive assessment of health disparities in tribal and non-tribal population in India: a sex- and age-stratified cross-sectional study

**DOI:** 10.3389/fpubh.2026.1772205

**Published:** 2026-04-13

**Authors:** Yashwant Kumar Ratre, Bhagirath Dave, Madhvi Joshi, Snehal Bagatharia, Tejas Shah, Sanman Samova, Chaitanya Joshi, Amrutlal Patel, Bhumika Prajapati

**Affiliations:** Gujarat Biotechnology Research Center, DST, Government of Gujarat, Gandhinagar, Gujarat, India

**Keywords:** anemia, glycemic risk, health disparities, micronutrients, tribal

## Abstract

**Background:**

Health disparities and glycemic risk among tribal and non-tribal populations in India remain poorly characterized particularly in the context of socio-demographic, behavioral, cultural, and clinical aspects. This study aimed to comprehensively assess various health parameters and to evaluate clinical predictors associated with cardiovascular burden to assess glycemic status in tribal and non-tribal adults.

**Methods:**

We conducted a community-based cross-sectional study across 33 districts of Gujarat and Madhya Pradesh. Participants were stratified by sex and age. Anthropometric measurements, blood pressure, and biochemical parameters including HbA1c, lipid profile, liver and renal function tests, and micronutrients were assessed. For comparison between groups non-parametric Mann–Whitney *U*-test and chi-square analysis was done. Multinomial logistic regression was applied to identified associations between clinical indicators and glycemic status. ROC curves was used to evaluate predictive accuracy of clinical indicators.

**Results:**

Among 1,720 participants, significant socio-demographic and behavioral differences were noted, with notably higher illiteracy rates (47.6%) and increased tobacco (30.3%) and alcohol consumption (8.4%) among tribal populations (all *p* < 0.001). Tribal individuals reflect considerably lower anthropometric measurements, blood pressure, fasting glucose, HbA1c, total cholesterol (TC), triglycerides (TG), and LDL cholesterol (all *p* < 0.05), as well as reduced hemoglobin levels but elevated red blood cell counts and eosinophils (*p* < 0.001), indicating a unique metabolic-hematologic profile. Further, multinomial regression analysis shown age as the significant predictor of dysglycemia in both groups, with increased odds of diabetic HbA1c levels in older tribal participants (*OR* = 5.830, *p* = 0.005) and non-tribal (*OR* = 28.252, *p* < 0.001); systolic hypertension was an independent predictor of diabetic HbA1c among tribal individuals (*OR* = 5.530, *p* = 0.015), while obesity (*OR* = 3.652, *p* < 0.001) and hypertriglyceridemia (*OR* = 4.690, *p* < 0.001) were notable correlates in non-tribal individuals. ROC analysis indicated acceptable predictive accuracy of age for glycemic risk i.e., diabetic HbA1c level (*AUC* = 0.687 tribal; 0.763 non-tribal) and systolic blood pressure (*AUC* = 0.725 tribal; 0.664 non-tribal).

**Conclusion:**

Tribal and non-tribal populations exhibit distinct sex- and age-specific health patterns, reflecting the coexistence of persistent undernutrition and an ongoing cardiovascular transition. These observations highlight the importance of developing population-specific, age-stratified screening frameworks and targeted prevention strategies to address emerging metabolic risk across diverse community settings. Such tailored approaches are essential to promote equitable and precision-oriented public health strategies.

## Introduction

Health disparities between tribal and non-tribal populations in India remain a persistent public health challenge, reflecting a complex interplay of undernutrition, emerging non-communicable diseases (NCDs), and micronutrient deficiencies. Alterations in various clinical parameters including, anthropometric and biochemical, contribute to this dual burden, increasing long-term health risks. Rapid demographic, nutritional, and lifestyle transitions have increased the prevalence of cardiovascular disorders including overweight, central obesity, type 2 diabetes, dyslipidemia, and cardiovascular disease as recognized by national surveys and the ICMR-INDIAB program (1). Recent national and regional studies further demonstrate that advancing age, elevated body mass index, hypertension, and dyslipidemia independently contribute to glycemic progression underscoring increased cardiovascular burden in Indian populations (2). At the same time, anemia, micronutrient deficiencies, and undernutrition continue to disproportionately impact maternal and child health (3). Emerging liver- and kidney-related metabolic conditions further complicate the health status. A recent meta-analysis found that nearly one-third of Indian adults have non-alcoholic fatty liver disease. In contrast, chronic kidney disease affects about 13% global population, with substantial regional variations across the country (4). Further, hypovitaminosis D (50–90%) and other micronutrient deficits exacerbate metabolic and immune dysregulation, underscoring the need for integrative biomarker surveillance and context-specific interventions (5).

India's tribal population exceeds 104 million, accounting for 8.6% of the national population (6). Tribal communities, spread across diverse ecological and cultural regions, face persistent socioeconomic disparity including poverty, low literacy, limited healthcare access, and marginal participation in mainstream development. Infectious diseases such as malaria, tuberculosis, and parasitic infections remain prevalent due to poor sanitation and geographical isolation, while NCDs such as hypertension, diabetes, and cardiovascular disorders are increasingly emerging (7). NFHS-5 data highlight particularly high burdens of malnutrition and anemia, with anemia prevalence among tribal women aged 15–49 years reaching 65.6% in certain states, compared to a national average of 57.2% (8). Chronic undernutrition is also evident in stunting and low BMI among tribal children and adults (9). However, the government has multiple programmes including the School Health and Wellness Programme (SHWP) and Rashtriya Kishor Swasthya Karyakram (RKSK) to focus on the overall development of tribal. Despite this, they remain far from mainstream development compared to non-tribal populations, indicating a need for further efforts. Although the overall burden of diabetes appears to be rising across communities, emerging evidence from studies indicates that sociodemographic groups exhibit distinct patterns and magnitudes of association among adiposity, blood pressure, lipid indices, and glycaemic status which suggest that cardiovascular burden architecture is not uniform across populations but varies according to underlying demographic, environmental, and genetic contexts (10). In contrast, non-tribal populations exhibit rising rates of overweight, obesity, dyslipidemia, and diabetes, largely driven by urbanization, sedentary lifestyles, and energy-dense diets (11). A comprehensive meta-analysis reported that the prevalence of diabetes in urban India increased from 3.3% in 1972 to 19.0% in 2015–2019, with urban populations exhibiting higher rates of obesity and dyslipidemia compared to their rural counterparts (12). The importance of age-stratified and community-specific risk modeling approaches is further reinforced by large-scale analyses of nationally representative datasets, including the Longitudinal Ageing Study in India. Findings from these studies demonstrate that advancing age, overweight or obesity, elevated systolic blood pressure, and hypertriglyceridemia independently predict glycemic status (13). Despite these insights, the existing literature is often fragmented, region-specific, or limited to a single health domain. Consequently, understanding of health disparities across the life course remains limited which restricting the development of targeted, community-specific interventions. Moreover, relatively few studies have systematically evaluated the predictive accuracy and discriminatory capacity of clinical indicators associated with cardiovascular burden in tribal compared with non-tribal populations (14). Therefore, a rigorous assessment of discrimination metrics is essential to determine whether commonly measured variables such as age, systolic blood pressure, body mass index, triglycerides, high-density lipoprotein (HDL) cholesterol, and low-density lipoprotein (LDL) cholesterol possess sufficient predictive performance to inform effective and context-specific screening strategies.

The present study addresses this critical gap by systematically comparing sociodemographic, anthropometric, biochemical, hematological, glycaemic, lipid, hepatic, renal, and micronutrient profiles between tribal and non-tribal populations. Further, this comprehensive evaluation is intended to generate a more refined understanding of glycaemic risk across socioeconomically diverse communities and to strengthen existing risk stratification frameworks. By contextualizing these findings within the framework of the Genome India Initiative, the study contributes to the advancement of precision public health and informs equity-oriented strategies aimed at reducing health disparities in India.

## Material and methods

### Study design and ethical approval

**Figure 1:**
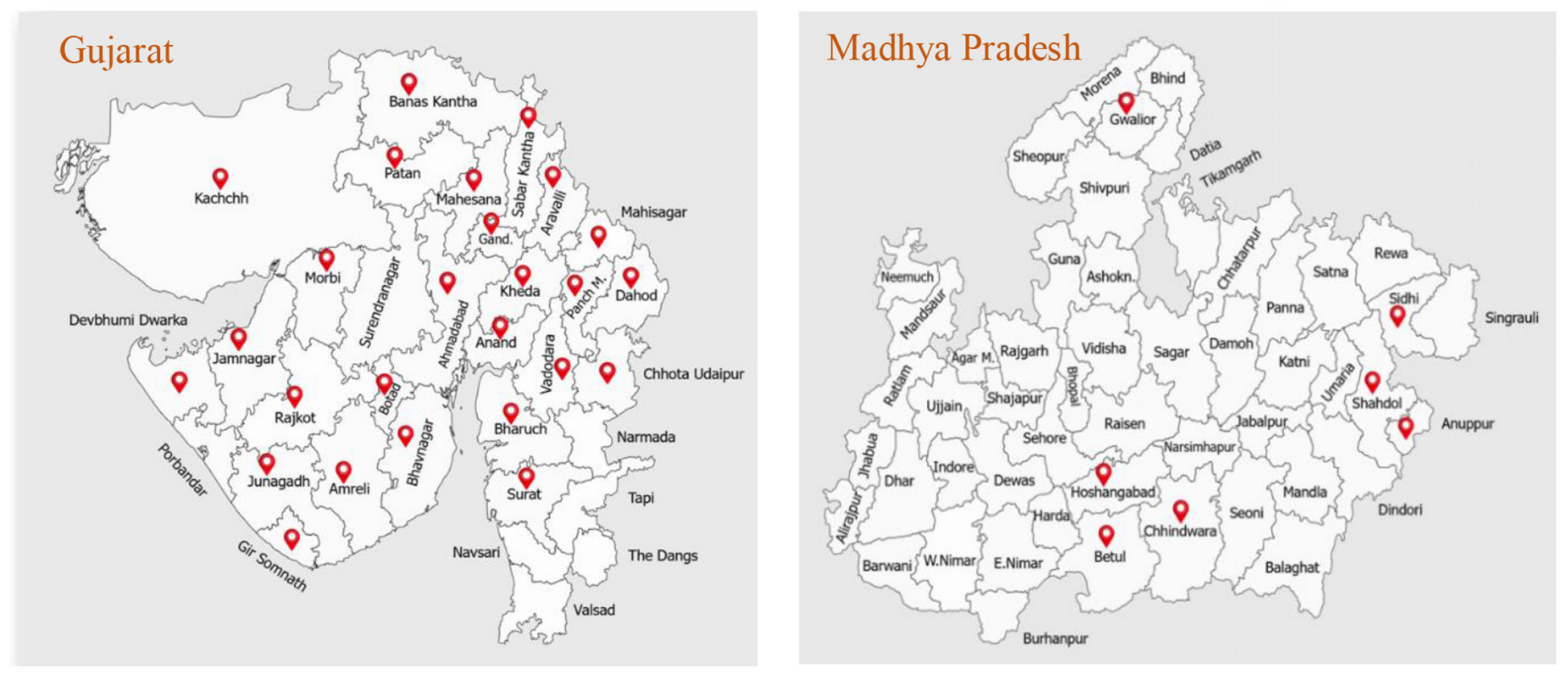
Sampling locations across districts of Gujarat and Madhya Pradesh included in the study.

This community-based cross-sectional study was conducted under the Genome India Initiative, supported by the Department of Biotechnology (DBT), Government of India, to investigate sex- and age-specific health disparities at the community level. The study was carried out between April 2020 and March 2023 across 33 districts in the states of Gujarat (*n* = 26) and Madhya Pradesh (*n* = 7), encompassing both tribal and non-tribal populations (Figure 1). Participants were recruited from ten socio-ethnically diverse communities, including five tribal and five non-tribal groups. Recruitment was facilitated through engagement with local communities at the block level. Written informed consent was obtained from all participants prior to enrollment and blood sample collection. The study protocol was approved by the Institutional Ethics Committee of the Gujarat Biotechnology Research Centre (GBRC), Department of Science and Technology, Government of Gujarat (Ref: GBRC/Ethics/GenomeIndia/2021-22).

### Study participants and sample size

A total of 1,720 self-reported healthy adults (≥18 years), including both tribal (*n* = 763) and non-tribal (*n* = 957) males and females, were enrolled in this study and stratified by age: young adults (18–39 years), middle-aged adults (40–59 years), and older (≥60 years). A random sampling approach was employed to ensure balanced representation of tribal and non-tribal populations. Participants were screened to confirm eligibility and overall health status prior to inclusion. Although the initial cohort comprised 1,720 individuals, the availability of data varied across specific parameters; therefore, analyses were performed using available data for each health variable (Table 2). The following criteria were applied before sample collection.

### Inclusion criteria:

- Adults aged ≥18 years.
- Self-reported healthy individuals not undergoing treatment for chronic illnesses.

### Exclusion criteria:

- Individuals with medical contraindications to venipuncture.
- Persons with terminal illness or those who were bedridden.

### Data collection and anthropometric measurements

Data were collected through a structured questionnaire administered by trained field workers. Information collected included sociodemographic details, and various health-related parameters. Anthropometric parameters were measured twice and the average value was used to minimize intra-observer variation. All assessments were performed by trained personnel using calibrated instruments.

### Anthropometric measurements were recorded using standardized protocols:

- Height: Measured in the standing position using a measuring tape.
- Weight: Assessed using a digital scale (±100 g precision) weighing balance (HBF-375, Omron Healthcare India Pvt. Ltd, Gurugram, Haryana, India), with participants after removing footwear.
- Waist Circumference: Waist circumference was assessed by placing a non-elastic measuring tape around the midpoint between the lowest rib and the top of the hip bone (iliac crest).
- Head Circumference: Recorded at the broadest part of the occiput and forehead using a flexible measuring tape.
- BMI: Body Mass Index was determined by dividing weight in kilograms (kg) by height in meters squared (m^2^).

### Blood pressure measurement

Systolic and diastolic blood pressure were recorded using a digital sphygmomanometer (HEM-7130-L, Omron Healthcare India Pvt. Ltd). Readings were taken on the left arm after a minimum rest of 5 min. Two readings were recorded at a 5-min interval, and the mean value was used for analysis.

### Biochemical and clinical laboratory analysis

Blood was drawn by certified phlebotomists into EDTA tubes (for glycated hemoglobin/HbA1c analysis) and clot-activator tubes (for serum biochemical measurements). All samples were maintained under cold-chain conditions and transported promptly to the sterling accuris pathology laboratory, Gujarat, India. Biochemical markers such as total cholesterol (TC), triglycerides (TG), high-density lipoprotein (HDL), and low-density lipoprotein (LDL) were measured using automated biochemical analyzers (Beckman Coulter AU680, Beckman Coulter, Inc.). HbA1c was measured using a high-performance liquid chromatography (HPLC)-based system (Tosoh HLC-723 G11, Tosoh Corporation, Tokyo, Japan), calibrated according to National Glycohemoglobin Standardization Program (NGSP) guidelines. All analyses were performed under standardized laboratory conditions to minimize variability.

### Definition and classification of clinical indicators

The definitions and classifications of cardiovascular clinical indicators utilized in this study were derived from established guidelines. Participants were stratified into three age categories young adults (18–39 years), middle-aged adults (40–59 years), and older adults (≥60 years) (15). Body mass index (BMI) was categorized using recommended cut-off values, classifying individuals as underweight (< 18.4 kg/m^2^), normal weight (18.5–22.9 kg/m^2^), overweight (23.0–24.9 kg/m^2^), or obese (≥25.0 kg/m^2^) (16). Glycemic status was determined using glycated hemoglobin (HbA1c) and categorized into non-diabetic (< 5.7%), prediabetic (5.7–6.4%), and diabetic (>6.5%) groups according to diagnostic thresholds (17). Blood pressure classification was based on systolic (SBP) and diastolic blood pressure (DBP) measurements. Normotension was defined as SBP < 120 mmHg and DBP < 80 mmHg; prehypertension as SBP 120–139 mmHg and DBP 80–89 mmHg; and hypertension as SBP ≥ 140 mmHg and DBP ≥ 90 mmHg respectively (18). Serum lipid parameters were categorized according to standard clinical cut-offs. Total cholesterol was classified as desirable (< 200 mg/dl), borderline high (200–239 mg/dl), or high (≥240 mg/dl). Triglycerides (mg/dl) were grouped as desirable (< 150 mg/dl), borderline high (150–199 mg/dl), or high (≥200 mg/dl). High-density lipoprotein (HDL) levels were categorized as low (< 40 mg/dl), intermediate (40–59 mg/dl), or high (≥60 mg/dl). Low-density lipoprotein (LDL) concentrations were defined as optimal (< 100 mg/dl), near or above optimal (100–129 mg/dl), borderline high (130–159 mg/dl), or high (≥160 mg/dl), following standard lipid management guidelines (18). All classification criteria were applied uniformly across the study population to ensure methodological consistency and comparability in the assessment of cardiovascular clinical indicators.

### Statistical analysis

Data were analyzed using IBM SPSS Statistics software (version 27.0). Descriptive statistics were employed to summarize socio-demographic, anthropometric, and biochemical parameters. Continuous variables were presented as mean ± standard deviation (SD), while categorical variables were expressed as percentages. The normality of continuous variables was assessed using the Kolmogorov-Smirnov test. For comparison between groups, non-parametric Mann-Whitney U-test and chi-square analyses were performed. Association between categorical variables, including socio-demographic characteristics, blood pressure status, BMI categories, and glycemic status, were evaluated using the Chi-square test. To investigate the relationship between clinical indicators associated with cardiovascular burden and glycemic status, multinomial logistic regression analyses were performed, and adjusted odds ratios (ORs) with corresponding confidence intervals were reported. Separate regression models were developed for tribal and non-tribal populations to identify distinct community-specific risk associations. Receiver operating characteristic (ROC) curve analysis was employed to evaluate the accuracy and discriminatory performance of clinically significant indicators to predict glycemic risk.

## Results

### Socio-demographic analysis of study population

A total of 1,720 individuals were included in the study, comprising 763 (44.4%) tribal and 957 (55.6%) non-tribal participants (Table 1). Significant differences were observed between the tribal and non-tribal groups across education level, marital status, and lifestyle behaviors. Educational levels varied markedly (*p* < 0.001). Nearly half of the tribal participants (47.6%) were illiterate compared with 10.1% among the non-tribal group. The proportion of graduates was considerably lower among tribals (3.1%) than non-tribals (22.5%), while 1.3% of tribals and 17.1% of non-tribals had completed postgraduate education. Conversely, a higher proportion of tribal participants had completed middle school (15.9%) compared to non-tribals (8.6%). Marital status distribution indicated that the majority of participants were currently married 84.9% among tribals and 79.7% among non-tribals. The proportion of never-married individuals was 13.5% in the tribal group and 18.0% in the non-tribal group, while widowed participants constituted 1.6% and 1.7%, respectively. A small but statistically significant difference was noted in the divorced/separated category (0% in tribals vs. 0.6% in non-tribals, *p* = 0.009). Lifestyle behavior analysis also revealed significant group differences. Current smoking was reported by 9.0% of tribals and 3.2% of non-tribals (*p* < 0.001), whereas most participants in both groups had never smoked (89.6% and 94.7%, respectively). Current tobacco chewing was more prevalent among tribals (30.3%) than non-tribals (15.8%) (*p* < 0.001). Similarly, current alcohol consumption was higher among tribals (8.4%) compared to non-tribals (3.2%) (*p* < 0.001). The majority reported never consuming alcohol (89.9% of tribals and 94.9% of non-tribals).

**Table 1:** Comparison of socio-demographic characteristics between tribal and non-tribal populations.

Socio-demographic characteristics		Tribal	Non-tribal	Total	*p*-value
Education level	Illiterate	363 (47.6)	97 (10.1)	460 (26.7)	< 0.001
	literate	101 (13.2)	123 (12.9)	224 (13)	
	Middle school	121 (15.9)	82 (8.6)	203 (11.8)	
	High school	102 (13.4)	161 (16.8)	263 (15.3)	
	Higher secondary	42 (5.5)	115 (12)	157 (9.1)	
	Graduate	24 (3.1)	215 (22.5)	239 (13.9)	
	Post graduate	10 (1.3)	164 (17.1)	174 (10.1)	
Marital status	Never married	103 (13.5)	172 (18)	275 (16)	0.009
	Currently married	648 (84.9)	763 (79.7)	1,411 (82)	
	Divorced/separated	0 (0)	6 (0.6)	6 (0.3)	
	Widowed	12 (1.6)	16 (1.7)	2 (1.6)	
Smoking status	Never	684 (89.6)	906 (94.7)	1,590 (92.4)	< 0.001
	Current	69 (9)	31 (3.2)	100 (5.8)	
	Past	10 (1.3)	20 (2.1)	30 (1.7)	
Tobacco chewing status	Never	526 (68.9)	786 (82.1)	1,312 (76.3)	< 0.001
	Current	231 (30.3)	151 (15.8)	382 (22.2)	
	Past	6 (0.8)	20 (2.1)	26 (1.5)	
Alcohol consumption status	Never	686 (89.9)	908 (94.9)	1,594 (92.7)	< 0.001
	Current	64 (8.4)	31 (3.2)	95 (5.5)	
	Past	13 (1.7)	18 (1.9)	31 (1.8)	

### Distribution of health parameters across tribal and non-tribal populations

This study assessed overall, sex- and age-specific distributions of health parameters between tribal and non-tribal populations. Analyses revealed consistent and significant disparities across anthropometric, hemodynamic, hematological, blood glucose, lipid, hepatic, renal, and micronutrient domains.

The mean age of tribal participants was significantly lower than that of non-tribals (*p* < 0.001). Anthropometric indices including waist and hip circumferences, height, weight, and BMI were consistently lower among tribals (*p* < 0.001 for all comparison). Both systolic and diastolic blood pressures were lower in tribals (*p* < 0.05). Metabolic markers showed lower fasting glucose and HbA1c (*p* < 0.001), with random blood sugar also differing between groups (*p* = 0.001). Hemoglobin levels were lower in tribals (*p* < 0.001), whereas red blood cell counts were higher (*p* < 0.001). White blood cell and platelet counts were lower, while eosinophils were markedly high in tribals (*p* < 0.001). Lipid parameters indicated lower total cholesterol, triglycerides, and LDL, with slightly higher HDL (*p* < 0.05). Liver function tests revealed higher SGPT and SGOT but lower alkaline phosphatase (*p* < 0.05 for all comparison) in tribal individuals. Micronutrient analysis showed higher vitamin B12 (*p* < 0.001), comparable vitamin D, and lower folic acid and homocysteine levels among tribals (*p* < 0.05; Table 2).

**Table 2:** Comparison of health parameters between tribal and non-tribal populations.

Health parameters	Community	Total (*N*)	Mean ±*SD*	*p*-value
Age (year)	Tribal	763	38.05 ± 13.36	< 0.001
	Non-tribal	957	41.84 ± 14.32	
Anthropometric parameters				
Waist circumference (cm)	Tribal	763	77.11 ± 8.49	< 0.001
	Non-tribal	957	83.44 ± 13.52	
Hip circumference (cm)	Tribal	763	84.49 ± 8.98	< 0.001
	Non-tribal	957	90.94 ± 14.40	
Height (cm)	Tribal	763	160.09 ± 7.79	< 0.001
	Non-tribal	957	164.51 ± 9.45	
Weight (kg)	Tribal	763	55.99 ± 10.72	< 0.001
	Non-tribal	957	65.49 ± 14.12	
BMI (kg/m^2^)	Tribal	763	21.84 ± 3.85	< 0.001
	Non-tribal	957	24.21 ± 5.02	
Hemodynamic parameters				
Systolic BP (mm Hg)	Tribal	763	127.61 ± 19.30	< 0.001
	Non-tribal	957	132.20 ± 19.99	
Diastolic BP (mm Hg)	Tribal	763	83.90 ± 12.24	0.015
	Non-tribal	957	85.15 ± 11.50	
Hematological parameters				
Hb (gm%)	Tribal	763	12.77 ± 2.03	< 0.001
	Non-tribal	957	13.42 ± 1.80	
Total RBC (million/c.mm)	Tribal	763	5.19 ± 0.72	< 0.001
	Non-tribal	957	4.84 ± 0.59	
Total WBC (cu/mm)	Tribal	762	6,728.53 ± 2,027.02	< 0.001
	Non-tribal	957	7,238.58 ± 1,803.77	
Platelet Count (per cu.mm)	Tribal	763	261,701.18 ± 82,655.90	< 0.001
	Non-tribal	957	293,941.48 ± 75,764.44	
Neutrophils %	Tribal	763	50.78 ± 10.09	< 0.001
	Non-tribal	957	53.17 ± 9.47	
Lymphocytes %	Tribal	763	35.90 ± 9.24	0.487
	Non-tribal	957	36.01 ± 8.72	
Eosinophils %	Tribal	763	5.29 ± 5.55	< 0.001
	Non-tribal	956	2.99 ± 2.76	
Monocytes %	Tribal	763	7.97 ± 3.37	0.152
	Non-tribal	957	7.65 ± 3.01	
Basophils %	Tribal	219	0.21 ± 0.16	< 0.001
	Non-tribal	688	0.26 ± 0.20	
Lipid parameters				
S. cholesterol (mg/dl)	Tribal	762	172.11 ± 39.17	< 0.001
	Non-tribal	957	188.97 ± 40.42	
S. triglyceride (mg/dl)	Tribal	761	125.79 ± 82.32	< 0.001
	Non-tribal	956	168.62 ± 106.31	
S. HDL (mg/dl)	Tribal	762	48.56 ± 16.63	0.010
	Non-tribal	957	45.54 ± 12.35	
S. LDL (mg/dl)	Tribal	762	98.57 ± 30.22	< 0.001
	Non-tribal	957	115.99 ± 34.73	
Hepatic parameters				
SGPT (U/L)	Tribal	762	31.12 ± 23.17	0.019
	Non-tribal	957	28.48 ± 19.12	
SGOT (U/L)	Tribal	762	37.28 ± 23.61	< 0.001
	Non-tribal	957	28.91 ± 12.10	
ALP (U/L)	Tribal	762	88.21 ± 30.50	< 0.001
	Non-tribal	957	93.98 ± 35.82	
Total bilirubin (mg/dl)	Tribal	762	0.55 ± 0.31	0.293
	Non-tribal	956	0.55 ± 0.29	
Direct bilirubin (mg/dl)	Tribal	757	0.19 ± 0.12	0.038
	Non-tribal	956	0.19 ± 0.08	
Indirect bilirubin (mg/dl)	Tribal	762	0.34 ± 0.23	0.003
	Non-tribal	956	0.35 ± 0.23	
Renal parameters				
Blood urea (mg/dl)	Tribal	762	25.06 ± 7.87	0.001
	Non-tribal	957	26.23 ± 7.26	
S. creatinine (mg/dl)	Tribal	762	0.75 ± 0.20	< 0.001
	Non-tribal	957	0.89 ± 0.24	
Blood glucose parameters				
RBS (mg/dl)	Tribal	397	127.89 ± 34.25	0.001
	Non-tribal	834	126.24 ± 42.63	
HbA1c (% total Hb)	Tribal	756	5.51 ± 0.87	< 0.001
	Non-tribal	957	5.89 ± 1.25	
Fasting blood glucose (mg/dl)	Tribal	755	112.11 ± 33.06	< 0.001
	Non-tribal	957	122.38 ± 35.88	
Micronutrient parameter				
Vitamin B12 (pg/ml)	Tribal	762	328.73 ± 206.50	< 0.001
	Non-tribal	920	254.22 ± 159.80	
Vitamin D (ng/ml)	Tribal	762	25.48 ± 8.33	0.152
	Non-tribal	921	24.89 ± 9.81	
Folic acid (ng/ml)	Tribal	760	6.88 ± 3.97	0.001
	Non-tribal	921	7.44 ± 4.14	
Homocysteine (mm/L)	Tribal	760	26.26 ± 12.38	0.025
	Non-tribal	921	27.96 ± 13.53	

### Gender-wise analysis of health parameters in tribal and non-tribal groups

Tribal men and women were younger than their non-tribal counterparts and exhibited lower anthropometric indices, including BMI, weight, height, waist, and hip circumferences (*p* ≤ 0.002 for all comparison). Blood pressure differences were modest: systolic pressure was significantly lower in tribal men (*p* < 0.001) but not in women, while diastolic pressure showed a mild decrease in men (*p* = 0.044). Hematological assessments indicated significantly lower hemoglobin levels in tribal participants (*p* < 0.001) accompanied by higher red blood cell counts (*p* < 0.001), whereas white blood cell and platelet counts were consistently low (*p* < 0.001). Neutrophil counts were lower (*p* = 0.002), while eosinophils were markedly high (*p* < 0.001). Lipid profiles were more favorable in tribal groups, with lower total cholesterol, triglycerides, and LDL cholesterol (*p* < 0.001 for all comparison), and higher HDL cholesterol observed in men (*p* < 0.001). Renal and hepatic markers demonstrated lower serum creatinine in both sexes (*p* < 0.001), high SGOT in both sexes (*p* < 0.001), and higher SGPT confined to tribal women (*p* < 0.001). Glycemic control was generally better in tribal populations, with significantly lower HbA1c and mean blood glucose (*p* < 0.001 for both comparison), although random glucose was slightly higher in tribal men (*p* = 0.009). Micronutrient profiles were heterogeneous: vitamin B12 levels were higher in both sexes (*p* < 0.001), folic acid was lower in men (*p* = 0.001), and vitamin D and homocysteine showed no significant differences (Table 3).

**Table 3:** Gender wise distribution of health parameters between tribal and non-tribal population.

Characteristics		Male			Female		
Health parameters	Community	Total (*N*)	Mean ±*SD*	*p*-value	Total (*N*)	Mean ±*SD*	*p*-value
Age (year)	Tribal	428	36.95 ± 13.31	< 0.001	335	39.45 ± 13.32	< 0.001
	Non-tribal	559	40.95 ± 15.00		398	43.10 ± 13.23	
Anthropometric parameters							
Waist circumference (cm)	Tribal	428	77.72 ± 8.49	< 0.001	335	76.32 ± 8.45	< 0.001
	Non-tribal	559	84.98 ± 12.68		398	81.28 ± 14.37	
Hip circumference (cm)	Tribal	428	84.90 ± 8.11	< 0.001	335	83.97 ± 9.97	< 0.001
	Non-tribal	559	91.47 ± 13.34		398	90.18 ± 15.76	
Height (cm)	Tribal	428	163.42 ± 6.80	< 0.001	335	155.83 ± 6.86	0.002
	Non-tribal	559	169.28 ± 6.94		398	157.81 ± 8.38	
Weight (kg)	Tribal	428	58.36 ± 10.71	< 0.001	335	52.98 ± 9.96	< 0.001
	Non-tribal	559	69.39 ± 13.46		398	60.00 ± 13.18	
BMI (kg/m^2^)	Tribal	428	21.87 ± 3.97	< 0.001	335	21.80 ± 3.70	< 0.001
	Non-tribal	559	24.22 ± 4.57		398	24.20 ± 5.60	
Hemodynamic parameters							
Systolic BP (mm Hg)	Tribal	428	128.35 ± 19.05	< 0.001	335	126.67 ± 19.61	0.076
	Non-tribal	559	134.12 ± 18.96		398	129.51 ± 21.08	
Diastolic BP (mm Hg)	Tribal	428	84.30 ± 12.63	0.044	335	83.37 ± 11.73	0.180
	Non-tribal	559	85.83 ± 11.83		398	84.20 ± 10.96	
Hematological parameters							
RBS (mg/dl)	Tribal	220	129.24 ± 38.83	0.009	177	126.22 ± 27.54	0.064
	Non-tribal	485	124.33 ± 36.07		349	128.89 ± 50.28	
Hb (gm%)	Tribal	428	13.79 ± 1.65	< 0.001	335	11.46 ± 1.69	< 0.001
	Non-tribal	559	14.39 ± 1.40		398	12.06 ± 1.37	
Total RBC (million/c.mm)	Tribal	428	5.45 ± 0.71	< 0.001	335	4.86 ± 0.59	< 0.001
	Non-tribal	559	5.05 ± 0.55		398	4.54 ± 0.50	
Total WBC (cu/mm)	Tribal	427	6,612.07 ± 2,077.29	< 0.001	335	6,876.99 ± 1,954.10	< 0.001
	Non-tribal	559	7,088.96 ± 1,831.04		398	7,448.72 ± 1,745.47	
Platelet Count (per cu.mm)	Tribal	428	253,250.00 ± 77,230.58	< 0.001	335	272,498.51 ± 88,050.91	< 0.001
	Non-tribal	559	279,631.48 ± 68,749.53		398	314,040.20 ± 80,537.35	
Neutrophils %	Tribal	428	50.54 ± 9.77	0.002	335	51.09 ± 10.49	< 0.001
	Non-tribal	559	52.57 ± 9.88		398	54.01 ± 8.80	
Lymphocytes %	Tribal	428	36.03 ± 9.23	0.665	335	35.73 ± 9.27	0.550
	Non-tribal	559	36.23 ± 9.23		398	35.69 ± 7.94	
Eosinophils %	Tribal	428	5.30 ± 5.42	< 0.001	335	5.27 ± 5.72	< 0.001
	Non-tribal	558	3.10 ± 2.87		398	2.83 ± 2.60	
Monocytes %	Tribal	428	8.06 ± 3.39	0.908	335	7.85 ± 3.36	0.038
	Non-tribal	559	7.92 ± 3.14		398	7.28 ± 2.77	
Basophils %	Tribal	122	0.22 ± 0.16	0.003	97	0.19 ± 0.17	< 0.001
	Non-tribal	403	0.27 ± 0.21		285	0.26 ± 0.17	
Lipid parameters							
S. cholesterol (mg/dl)	Tribal	427	171.93 ± 40.23	< 0.001	335	172.34 ± 37.84	< 0.001
	Non-tribal	559	189.85 ± 41.48		398	187.74 ± 38.91	
S. triglyceride (mg/dl)	Tribal	426	133.04 ± 90.03	< 0.001	335	116.59 ± 70.39	< 0.001
	Non-tribal	559	184.91 ± 113.37		397	145.70 ± 90.80	
S. HDL (mg/dl)	Tribal	427	48.00 ± 17.83	< 0.001	335	49.29 ± 14.96	0.152
	Non-tribal	559	42.71 ± 11.28		398	49.52 ± 12.70	
S. LDL (mg/dl)	Tribal	427	98.48 ± 30.72	< 0.001	335	98.67 ± 29.60	< 0.001
	Non-tribal	559	117.63 ± 35.23		398	113.69 ± 33.92	
Hepatic parameters							
SGPT (U/L)	Tribal	427	35.48 ± 26.79	0.542	335	25.56 ± 15.91	< 0.001
	Non-tribal	559	33.14 ± 21.59		398	21.93 ± 12.33	
SGOT (U/L)	Tribal	427	41.64 ± 28.67	< 0.001	335	31.72 ± 12.88	< 0.001
	Non-tribal	559	31.41 ± 12.86		398	25.39 ± 9.94	
ALP (U/L)	Tribal	427	88.56 ± 29.37	0.010	335	87.76 ± 31.92	0.005
	Non-tribal	559	93.99 ± 35.05		398	93.97 ± 36.92	
Total bilirubin (mg/dL)	Tribal	427	0.61 ± 0.34	0.869	335	0.47 ± 0.24	0.492
	Non-tribal	558	0.60 ± 0.31		398	0.48 ± 0.24	
Direct bilirubin (mg/dl)	Tribal	423	0.20 ± 0.11	0.002	334	0.19 ± 0.12	0.626
	Non-tribal	558	0.20 ± 0.08		398	0.17 ± 0.07	
Indirect bilirubin (mg/dl)	Tribal	427	0.38 ± 0.25	0.260	335	0.28 ± 0.18	0.007
	Non-tribal	558	0.39 ± 0.26		398	0.31 ± 0.19	
Renal parameters							
Blood urea (mg/dl)	Tribal	427	25.89 ± 8.16	0.179	335	24.00 ± 7.35	< 0.001
	Non-tribal	559	26.57 ± 7.40		398	25.77 ± 7.04	
S.Creatinine (mg/dl)	Tribal	427	0.85 ± 0.18	< 0.001	335	0.63 ± 0.13	< 0.001
	Non-tribal	559	0.99 ± 0.22		398	0.74 ± 0.18	
Blood glucose parameters							
RBS (mg/dl)	Tribal	220	129.24 ± 38.83	0.009	177	126.22 ± 27.54	0.064
	Non-tribal	485	124.33 ± 36.07		349	128.89 ± 50.28	
HbA1C (% Total Hb)	Tribal	422	5.57 ± 1.01	< 0.001	334	5.42 ± 0.65	< 0.001
	Non-tribal	559	5.90 ± 1.23		398	5.87 ± 1.28	
Fasting blood glucose (mg/dl)	Tribal	421	114.58 ± 40.95	< 0.001	334	108.99 ± 18.53	< 0.001
	Non-tribal	559	122.69 ± 35.21		398	121.94 ± 36.85	
Micronutrient parameters							
Vitamin B12 (pg/ml)	Tribal	427	327.42 ± 221.38	< 0.001	335	330.40 ± 186.12	< 0.001
	Non-tribal	537	238.89 ± 155.46		383	275.71 ± 163.49	
Vitamin D (ng/ml)	Tribal	427	26.95 ± 8.88	0.051	335	23.59 ± 7.15	0.869
	Non-tribal	537	25.86 ± 10.28		384	23.54 ± 8.96	
Folic acid (ng/ml)	Tribal	425	6.10 ± 3.57	0.001	335	7.86 ± 4.23	0.075
	Non-tribal	537	6.79 ± 3.88		384	8.36 ± 4.32	
Homocysteine (mm/L)	Tribal	425	31.75 ± 12.37	0.225	335	19.30 ± 8.18	0.096
	Non-tribal	537	32.81 ± 13.39		384	21.17 ± 10.47	

### Age-specific analysis of health parameters among tribal and non-tribal populations

Across all age groups, tribal populations exhibited lower anthropometric indices (*p* < 0.05 for all comparison) and lower hemoglobin (*p* < 0.05), while RBC counts remained high (*p* ≤ 0.001). The WBC and platelet counts were significantly lower in younger and middle-aged tribal adults (*p* ≤ 0.001). Lipid profile disparities persisted, with lower cholesterol, triglycerides, and LDL, and higher HDL observed in younger tribal adults (*p* < 0.001 for all comparison). Glycemic markers, including HbA1c and mean blood glucose, were lower in middle-aged and older tribal participants (*p* < 0.001 for both comparison). Renal function markers showed lower creatinine across all age groups (*p* < 0.001), while hepatic markers revealed high SGOT in all age groups (*p* < 0.001) and lower alkaline phosphatase and indirect bilirubin in younger and middle-aged adults (*p* < 0.05). Micronutrient assessments demonstrated higher vitamin B12 (*p* < 0.001), lower folic acid in younger adults (*p* < 0.001), slightly higher vitamin D in the youngest age group (*p* = 0.022), and lower homocysteine in middle-aged tribal participants (*p* = 0.002; Table 4).

**Table 4:** Age wise distribution of health parameters between tribal and non-tribal population.

Characteristics		Young adults (18–39 y)			Middle-aged adults (40–59 y)			Older (≥60 y)		
Health parameters	Community	*N*	Mean ±*SD*	*p*-value	*N*	Mean ±*SD*	*p*-value	*N*	Mean ±*SD*	*p*-value
Age (year)	Tribal	441	28.41 ± 5.89	0.093	259	47.88 ± 5.32	0.083	63	65.08 ± 4.95	0.350
	Non-tribal	448	29.14 ± 5.59		384	48.68 ± 5.60		125	66.36 ± 6.19	
Anthropometric parameters										
Waist circumference (cm)	tribal	441	75.41 ± 8.01	< 0.001	259	79.80 ± 8.30	< 0.001	63	77.87 ± 9.65	0.001
	Non-tribal	448	80.35 ± 12.46		384	86.44 ± 14.10		125	85.27 ± 13.06	
Hip circumference (cm)	Tribal	441	83.37 ± 8.61	< 0.001	259	86.60 ± 9.01	< 0.001	63	83.69 ± 9.97	< 0.001
	Non-tribal	448	88.20 ± 13.93		384	93.97 ± 14.56		125	91.38 ± 13.74	
Height (cm)	Tribal	441	160.32 ± 7.69	< 0.001	259	159.65 ± 7.91	< 0.001	63	160.21 ± 8.09	0.049
	Non-tribal	448	166.27 ± 8.72		384	162.95 ± 9.52		125	162.98 ± 10.62	
Weight (kg)	Tribal	441	55.67 ± 10.96	< 0.001	259	56.62 ± 10.49	< 0.001	63	55.62 ± 9.97	< 0.001
	Non-tribal	448	65.07 ± 14.10		384	66.56 ± 14.45		125	63.65 ± 12.94	
BMI (kg/m^2^)	Tribal	441	21.65 ± 3.98	< 0.001	259	22.19 ± 3.63	< 0.001	63	21.70 ± 3.73	< 0.001
	Non-tribal	448	23.50 ± 4.64		384	25.10 ± 5.40		125	24.06 ± 4.74	
Hemodynamic parameters										
Systolic BP (mm Hg)	Tribal	441	124.29 ± 16.44	0.134	259	129.23 ± 19.13	< 0.001	63	144.21 ± 27.72	0.345
	Non-tribal	448	125.54 ± 15.76		384	135.30 ± 19.81		125	146.57 ± 23.97	
Diastolic BP (mm Hg)	Tribal	441	82.70 ± 11.84	0.680	259	84.35 ± 11.40	< 0.001	63	90.41 ± 15.83	0.229
	Non-tribal	448	82.32 ± 10.47		384	87.89 ± 11.74		125	86.92 ± 11.96	
Hematological parameters										
Hb (gm%)	Tribal	441	12.93 ± 1.97	< 0.001	259	12.52 ± 2.12	< 0.001	63	12.61 ± 1.85	0.046
	Non-tribal	448	13.65 ± 1.85		384	13.25 ± 1.75		125	13.10 ± 1.68	
Total RBC (million/c.mm)	Tribal	441	5.18 ± 0.70	< 0.001	259	5.24 ± 0.74	< 0.001	63	5.01 ± 0.71	0.001
	Non-tribal	448	4.89 ± 0.57		384	4.84 ± 0.58		125	4.64 ± 0.64	
Total WBC (cu/mm)	Tribal	441	6,810.86 ± 2,116.72	0.001	259	6,602.94 ± 1,910.88	< 0.001	63	6,669.84 ± 1,842.57	0.003
	Non-tribal	448	7,111.65 ± 1,774.80		384	7,301.64 ± 1,803.37		125	7,499.76 ± 1,882.75	
Platelet count (per cu.mm)	Tribal	441	268,551.02 ± 82,299.44	< 0.001	259	253,173.75 ± 80,545.55	< 0.001	63	248,809.52 ± 89,980.44	0.114
	Non-tribal	448	307,392.86 ± 72,397.98		384	293,458.33 ± 77,904.66		125	268,672.00 ± 75,955.82	
Neutrophils %	Tribal	441	50.76 ± 9.68	0.017	259	50.63 ± 10.81	0.001	63	51.59 ± 9.95	0.019
	Non-tribal	448	52.41 ± 9.50		384	53.38 ± 9.41		125	55.21 ± 9.20	
Lymphocytes %	Tribal	441	36.18 ± 8.86	0.071	259	35.68 ± 9.82	0.759	63	34.79 ± 9.39	0.336
	Non-tribal	448	37.10 ± 8.47		384	35.54 ± 8.84		125	33.52 ± 8.62	
Eosinophils %	Tribal	441	5.27 ± 5.65	< 0.001	259	5.38 ± 5.64	< 0.001	63	5.01 ± 4.30	0.001
	Non-tribal	448	2.93 ± 2.96		383	3.08 ± 2.62		125	2.87 ± 2.39	
Monocytes %	Tribal	441	7.72 ± 3.39	0.332	259	8.25 ± 3.32	0.146	63	8.57 ± 3.30	0.272
	Non-tribal	448	7.38 ± 2.96		384	7.80 ± 2.88		125	8.16 ± 3.42	
Basophils %	Tribal	118	0.20 ± 0.16	0.014	84	0.22 ± 0.17	0.001	17	0.18 ± 0.10	0.022
	Non-tribal	282	0.25 ± 0.21		310	0.26 ± 0.17		96	0.30 ± 0.23	
Lipid parameters										
S. cholesterol (mg/dl)	Tribal	440	165.06 ± 37.51	< 0.001	259	181.68 ± 39.54	< 0.001	63	181.97 ± 39.08	0.113
	Non-tribal	448	179.01 ± 37.68		384	198.91 ± 40.78		125	194.14 ± 40.64	
S. triglyceride (mg/dl)	Tribal	440	115.06 ± 74.36	< 0.001	259	139.22 ± 83.07	< 0.001	63	145.40 ± 115.49	0.010
	Non-tribal	448	155.56 ± 105.03		384	181.46 ± 104.87		125	175.87 ± 110.57	
S. HDL (mg/dl)	Tribal	440	49.21 ± 17.15	< 0.001	259	47.67 ± 15.61	0.765	63	47.72 ± 17.03	0.727
	Non-tribal	448	44.57 ± 12.31		384	46.22 ± 12.01		125	46.90 ± 13.29	
S. LDL (mg/dl)	Tribal	440	92.12 ± 28.19	< 0.001	259	106.96 ± 31.15	< 0.001	63	109.05 ± 28.99	0.016
	Non-tribal	448	108.46 ± 33.51		384	123.24 ± 34.92		125	120.72 ± 33.06	
Hepatic parameters										
SGPT(U/L)	Tribal	440	32.51 ± 22.14	0.122	259	30.79 ± 26.55	0.559	63	22.71 ± 10.01	0.302
	Non-tribal	448	29.97 ± 20.59		384	28.51 ± 17.83		125	23.03 ± 16.44	
SGOT (U/L)	Tribal	440	39.40 ± 25.71	< 0.001	259	35.16 ± 21.49	< 0.001	63	31.12 ± 12.08	< 0.001
	Non-tribal	448	29.33 ± 12.56		384	29.36 ± 11.58		125	26.00 ± 11.62	
ALP (U/L)	Tribal	440	86.81 ± 32.45	0.013	259	89.47 ± 28.19	0.008	63	92.72 ± 24.66	0.827
	Non-tribal	448	94.41 ± 42.99		384	94.15 ± 28.40		125	91.88 ± 27.12	
Total bilirubin (mg/dl)	Tribal	440	0.57 ± 0.32	0.449	259	0.52 ± 0.29	0.226	63	0.53 ± 0.27	0.809
	Non-tribal	447	0.58 ± 0.32		384	0.52 ± 0.25		125	0.54 ± 0.26	
Direct bilirubin (mg/dl)	Tribal	436	0.18 ± 0.11	0.028	258	0.20 ± 0.13	0.660	63	0.19 ± 0.11	0.379
	Non-tribal	447	0.19 ± 0.08		384	0.18 ± 0.08		125	0.19 ± 0.08	
Indirect bilirubin (mg/dl)	Tribal	440	0.35 ± 0.24	0.019	259	0.31 ± 0.22	0.036	63	0.32 ± 0.18	0.415
	Non-tribal	447	0.38 ± 0.26		384	0.32 ± 0.20		125	0.34 ± 0.20	
Renal parameters										
Blood urea (mg/dl)	Tribal	440	24.27 ± 7.74	0.999	259	25.70 ± 7.77	0.009	63	27.92 ± 8.29	0.019
	Non-tribal	448	24.03 ± 6.05		384	27.11 ± 6.85		125	31.43 ± 9.05	
S. creatinine (mg/dl)	Tribal	440	0.74 ± 0.20	< 0.001	259	0.75 ± 0.18	< 0.001	63	0.80 ± 0.21	< 0.001
	Non-tribal	448	0.85 ± 0.21		384	0.90 ± 0.23		125	0.98 ± 0.32	
Blood glucose Parameters										
RBS (mg/dl)	Tribal	207	123.85 ± 33.43	< 0.001	153	129.90 ± 35.12	0.442	37	142.16 ± 31.36	0.348
	Non-tribal	366	115.30 ± 28.66		352	131.77 ± 45.79		116	143.93 ± 58.00	
HbA1C (% total Hb)	Tribal	437	5.40 ± 0.79	0.768	256	5.59 ± 0.92	< 0.001	63	5.87 ± 1.03	< 0.001
	Non-tribal	448	5.47 ± 0.80		384	6.15 ± 1.31		125	6.61 ± 1.74	
Fasting blood glucose (mg/dl)	Tribal	437	108.39 ± 22.75	0.792	256	116.11 ± 45.62	< 0.001	63	121.65 ± 29.46	< 0.001
	Non-tribal	448	110.28 ± 23.23		384	129.82 ± 37.60		125	142.86 ± 49.91	
Micronutrient parameters										
Vitamin B12 (pg/ml)	Tribal	440	316.73 ± 184.11	< 0.001	259	332.94 ± 216.25	< 0.001	63	395.25 ± 288.42	< 0.001
	Non-tribal	416	241.20 ± 161.12		379	259.79 ± 151.03		125	280.63 ± 177.38	
Vitamin D (ng/ml)	Tribal	440	25.85 ± 8.85	0.022	259	25.05 ± 7.26	0.789	63	24.62 ± 8.68	0.874
	Non-tribal	417	24.51 ± 10.46		379	25.32 ± 9.24		125	24.83 ± 9.27	
Folic acid (ng/ml)	Tribal	438	6.30 ± 3.72	< 0.001	259	7.65 ± 4.26	0.987	63	7.72 ± 3.82	0.839
	Non-tribal	417	7.03 ± 3.80		379	7.63 ± 4.20		125	8.28 ± 4.86	
Homocysteine (mm/L)	Tribal	438	28.42 ± 13.15	0.389	259	22.91 ± 10.34	0.002	63	25.04 ± 11.39	0.271
	Non-tribal	417	29.53 ± 14.30		379	26.60 ± 13.06		125	26.81 ± 11.73	

### Association between clinical indicators and glycemic status

To evaluate the association between clinical factors and glycemic status between tribal and non-tribal populations, we performed a multinomial logistic regression analysis (Table 5). Glycemic categories were defined based on HbA1c levels, with non-diabetes HbA1c level (reference) for this investigation. Within the tribal population, age was identified as the most significant predictor of glycemic progression. Specifically, older tribal individuals exhibited significantly greater odds of having prediabetic HbA1c levels (*OR* = 2.888, *p* = 0.001) and diabetic HbA1c levels (*OR* = 5.830, *p* = 0.005) when compared to younger adults. Likewise, in the non-tribal population, advanced age was strongly correlated with prediabetic (*OR* = 5.408, *p* < 0.001) and diabetic HbA1c levels (*OR* = 28.252, *p* < 0.001). Additionally, middle-aged individuals in the non-tribal group demonstrated significantly increased odds for both prediabetic (*OR* = 3.797, *p* < 0.001) and diabetic HbA1c levels (*OR* = 8.801, *p* < 0.001). In the tribal cohort, systolic hypertension was significantly linked to prediabetic (*OR* = 2.479, *p* = 0.002) and diabetic HbA1c levels (*OR* = 5.530, *p* = 0.015). Conversely, among non-tribal participants, systolic prehypertension was associated with diabetic HbA1c levels (*OR* = 2.864, *p* = 0.005). No statistically significant associations were found for diastolic blood pressure categories in either population. With respect to adiposity, obesity was significantly correlated with prediabetic (*OR* = 1.907, *p* = 0.001) and diabetic HbA1c levels (*OR* = 3.652, *p* < 0.001) in the non-tribal population. Furthermore, being overweight was associated with increased odds of prediabetic (*OR* = 1.790, *p* = 0.013) and diabetic HbA1c levels (*OR* = 2.168, *p* = 0.025) among non-tribal individuals. However, these associations did not show statistical significance among tribal participants. In terms of lipid parameters, increased triglyceride levels were linked to diabetic HbA1c levels in the non-tribal population (*OR* = 4.690, *p* < 0.001), and borderline triglyceride levels exhibited a similar association (*OR* = 2.068, *p* = 0.023). Conversely, in the tribal population, low HDL levels showed an inverse relationship with prediabetic HbA1c levels (*OR* = 0.541, *p* = 0.023), as did borderline HDL levels (*OR* = 0.549, *p* = 0.012). Furthermore, elevated LDL levels were correlated with prediabetic HbA1c levels among non-tribal individuals (*OR* = 2.312, *p* = 0.043), and borderline LDL levels were also significantly associated (*OR* = 2.275, *p* = 0.004). No statistically significant associations were found regarding gender in either population.

**Table 5:** Association between clinical indicators and glycemic status among tribal and non-tribal populations.

Clinical indicators	Classification	Tribal				Non-tribal			
		Pre-diabetic HbA1c level (5.7%-6.4)		Diabetic HbA1c level (>6.5%)		Pre-diabetic HbA1c level (5.7%-6.4)		Diabetic HbA1c level (>6.5%)	
		Odds ratio	*p*-value	Odds ratio	*p*-value	Odds ratio	*p*-value	Odds ratio	*p*-value
Gender	Male	1.214	0.288	2.363	0.075	0.919	0.623	1.292	0.281
	Female (ref)								
Age group	Older	2.888	0.001	5.830	0.005	5.408	< 0.001	28.252	< 0.001
	Middle-aged	1.227	0.303	2.620	0.050	3.797	< 0.001	8.801	< 0.001
	Young adults (ref)								
Systolic BP (mm Hg)	Hypertension	2.479	0.002	5.530	0.015	0.682	0.162	2.227	0.052
	Prehypertension	1.364	0.164	1.382	0.615	1.151	0.528	2.864	0.005
	Normal (Ref)								
Diastolic BP (mm Hg)	Hypertension	0.588	0.063	0.466	0.249	1.603	0.058	1.189	0.605
	Prehypertension	0.719	0.134	0.391	0.117	1.348	0.165	1.017	0.957
	Normal (ref)								
BMI (kg/m^2^)	Obesity	1.277	0.321	2.340	0.085	1.907	0.001	3.652	< 0.001
	Overweight	0.949	0.842	0.698	0.612	1.790	0.013	2.168	0.025
	Underweight	0.714	0.193	0.485	0.370	1.245	0.434	1.381	0.507
	Normal (ref)								
S. cholesterol (mg/dl)	Risk	0.640	0.409	1.846	0.476	0.380	0.018	0.405	0.080
	Borderline	0.976	0.939	1.620	0.472	0.652	0.074	0.554	0.072
	Desirable (ref)								
S. triglyceride (mg/dl)	High	1.289	0.360	2.896	0.051	1.406	0.128	4.690	< 0.001
	Borderline	1.310	0.330	1.226	0.777	1.311	0.230	2.068	0.023
	Desirable (ref)								
S. HDL (mg/dl)	Low	0.541	0.023	0.820	0.750	0.651	0.131	0.844	0.690
	Borderline	0.549	0.012	0.694	0.536	0.800	0.367	0.974	0.947
	High (ref)								
S. LDL (mg/dl)	High	2.267	0.174	0.697	0.736	2.312	0.043	1.443	0.497
	Borderline	1.403	0.366	0.529	0.420	2.275	0.004	1.001	0.997
	Above optimal	1.297	0.229	0.583	0.354	0.985	0.941	0.619	0.097
	Optimal (ref)								

### Predictive accuracy of clinical indicators to identify glycemic risk

**Figure 2:**
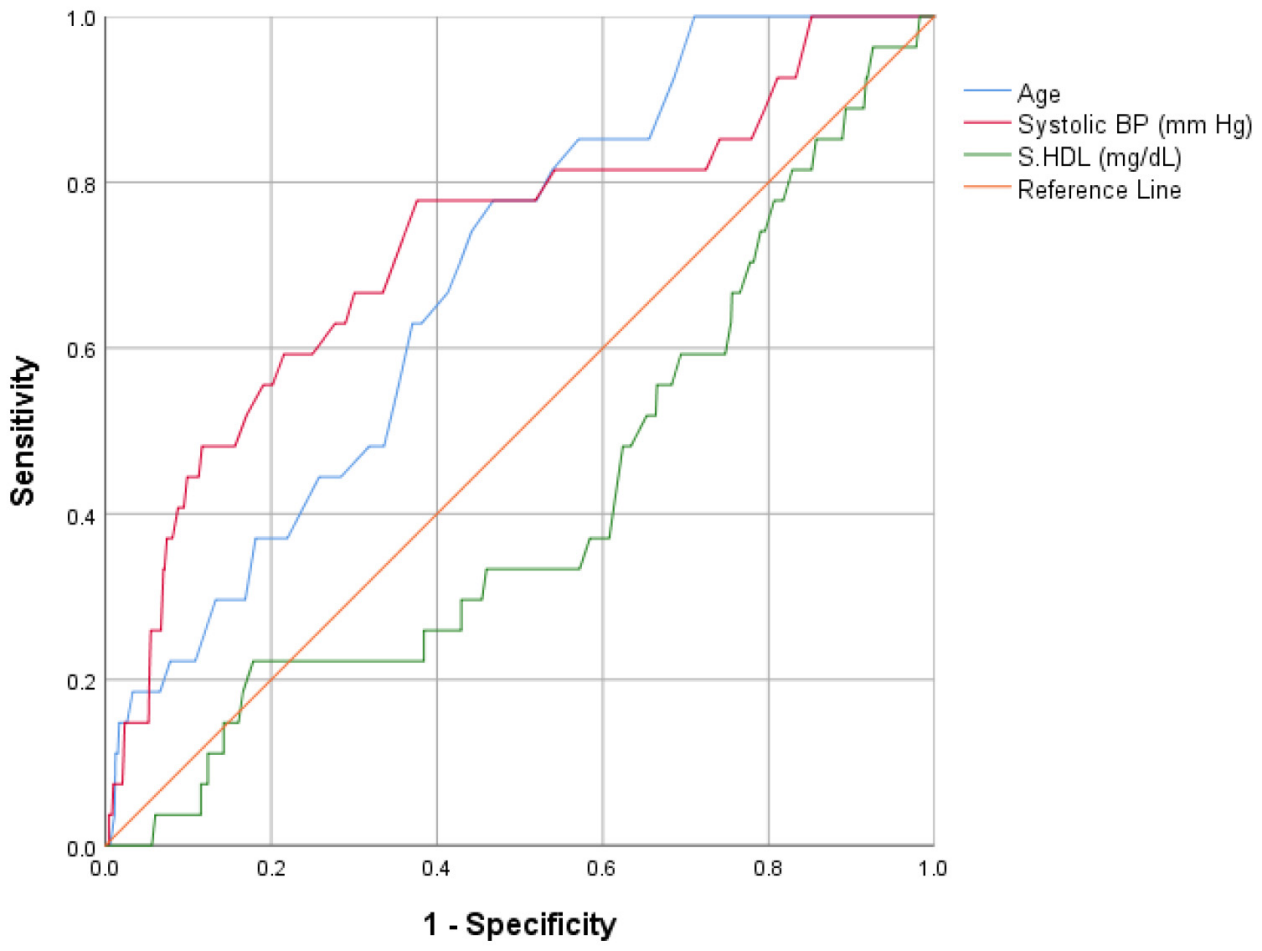
Clinical indicators for prediction of prediabetic HbA1c levels in tribal population.

**Figure 3:**
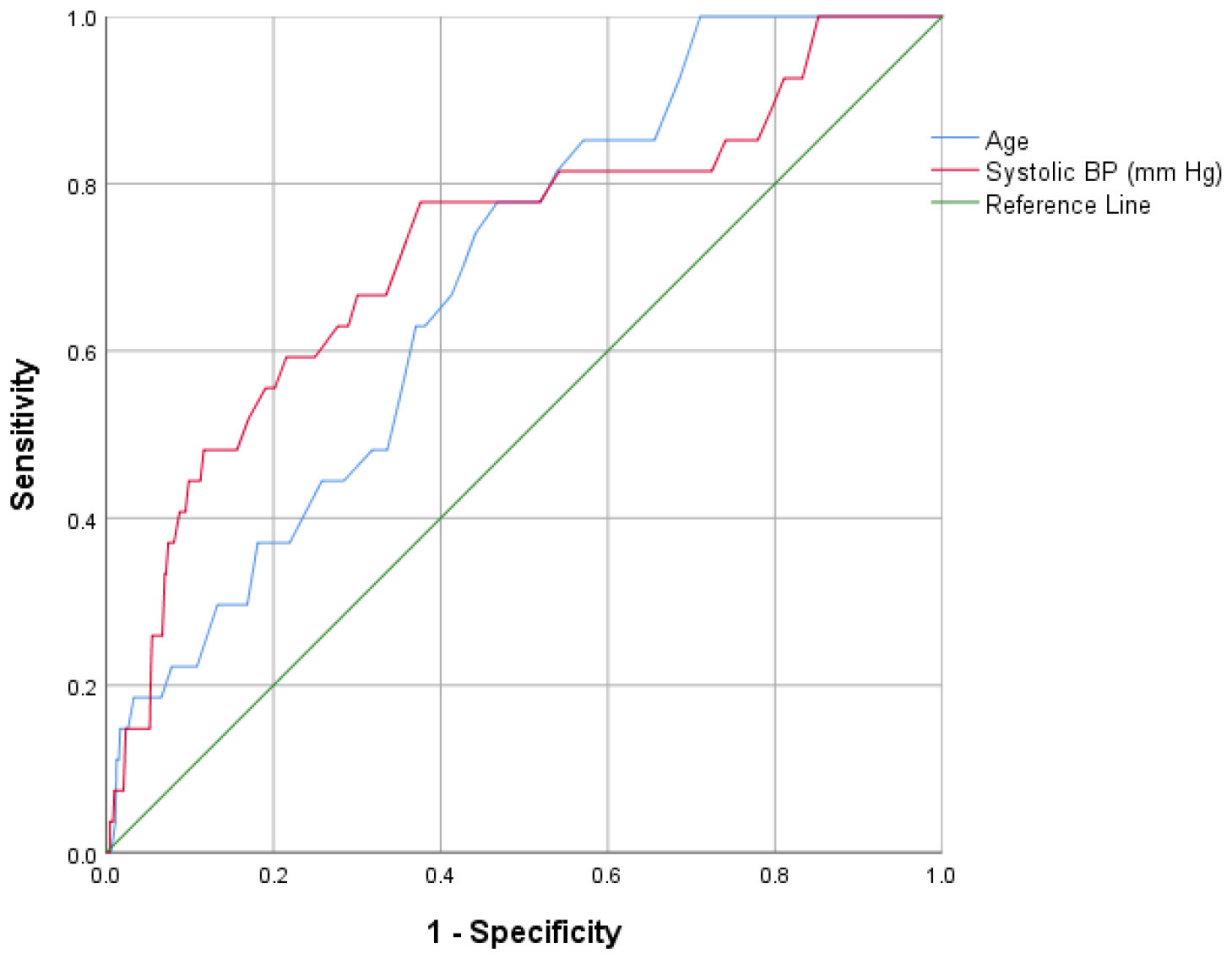
Clinical indicators for prediction of diabetic HbA1c levels in tribal population.

**Figure 4:**
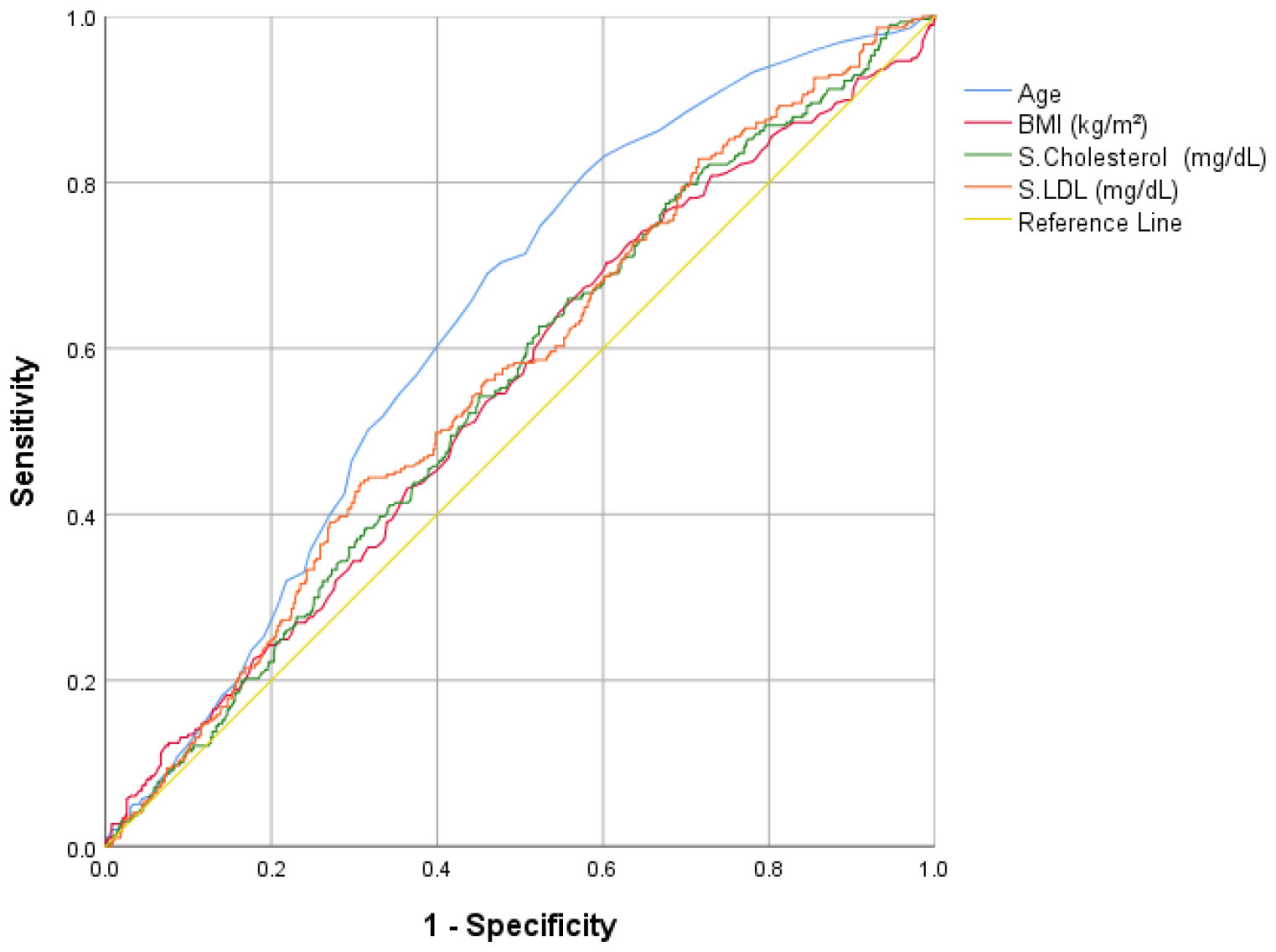
Clinical indicators for prediction of prediabetic HbA1c levels in non-tribal population.

**Figure 5:**
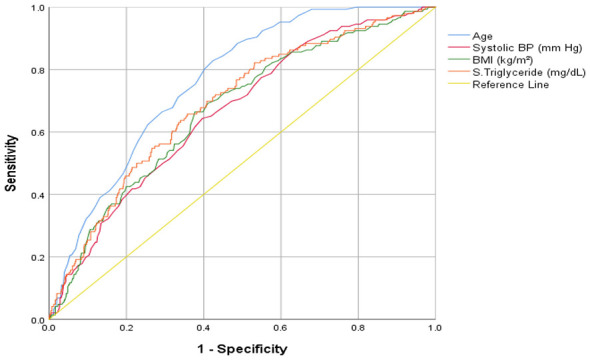
Clinical indicators for prediction of diabetic HbA1c levels in non-tribal population.

Receiver operating characteristic (ROC) curve analysis was performed to assess the discriminatory accuracy of cardiovascular clinical indicators that demonstrated significant associations in the multinomial regression analysis for predicting glycemic status among tribal and non-tribal populations (Table 6 and Figures 2–5). Among tribal individuals, age demonstrated modest discrimination for prediabetic HbA1c levels (*AUC* = 0.582, 95% *CI*: 0.533–0.631; *p* = 0.001) and acceptable discrimination for diabetic HbA1c levels (*AUC* = 0.687, 95% *CI*: 0.600–0.774; *p* = 0.001). Systolic blood pressure showed an *AUC* of 0.598 (95% CI: 0.551–0.646; *p* < 0.001) for prediabetic and 0.725 (95% *CI*: 0.617–0.832; *p* < 0.001) for diabetic HbA1c levels, indicating higher discriminatory ability than age for overt dysglycemia. HDL cholesterol exhibited limited predictive efficiency for prediabetic HbA1c (*AUC* = 0.545, 95% *CI*: 0.496–0.594; *p* = 0.064), and the association was not significant.

**Table 6:** Predictive accuracy of clinical indicators for abnormal glycemic status.

Clinical indicators	Tribal				Non-tribal			
	Pre-diabetic HbA1c level		Diabetic HbA1c level		Pre-diabetic HbA1c level		Diabetic HbA1c level	
	AUC (95% *CI*)	*p*-value	AUC (95% *CI*)	*p*-value	AUC (95% *CI*)	*p*-value	AUC (95% *CI*)	*p*-value
Age	0.582 (0.533–0.631)	0.001	0.687 (0.600–0.774)	0.001	0.631 (0.595–0.667)	< 0.001	0.763 (0.728–0.799)	< 0.001
Systolic BP (mm Hg)	0.598 (0.551–0.646)	< 0.001	0.725 (0.617–0.832)	< 0.001	–	-	0.664 (0.619–0.709)	< 0.001
BMI (kg/m^2^)	–	–	–	–	0.548 (0.508–0.587)	0.018	0.670 (0.624–0.717)	< 0.001
S. triglyceride (mg/dl)	–	–	–	–	–	–	0.689 (0.643–0.735)	< 0.001
S. cholesterol (mg/dl)	–	–	–	–	0.553 (0.514–0.591)	0.009	–	–
S. HDL (mg/dl)	0.545 (0.496–0.594)	0.064	–	–	–	–	–	–
S. LDL (mg/dl)	–	–	–	–	0.566 (0.527–0.604)	0.001	–	–

In the non-tribal population, age demonstrated stronger predictive performance, with an *AUC* of 0.631 (95% *CI*: 0.595–0.667; *p* < 0.001) for prediabetic and 0.763 (95% *CI*: 0.728–0.799; *p* < 0.001) for diabetic HbA1c level, reflecting good discrimination for overt hyperglycemia. BMI showed modest discrimination for prediabetic (*AUC* = 0.548, 95% *CI*: 0.508–0.587; *p* = 0.018) and moderate discrimination for diabetic HbA1c range (*AUC* = 0.670, 95% *CI*: 0.624–0.717; *p* < 0.001). Systolic blood pressure was significantly associated with diabetic HbA1c levels (*AUC* = 0.664, 95% *CI*: 0.619–0.709; *p* < 0.001). Triglycerides demonstrated moderate discrimination for diabetic HbA1c (*AUC* = 0.689, 95% *CI*: 0.643–0.735; *p* < 0.001). Total cholesterol and LDL cholesterol showed modest but significant discrimination for prediabetic HbA1c level (*AUC* = 0.553, 95% *CI*: 0.514–0.591; *p* = 0.009 and *AUC* = 0.566, 95% *CI*: 0.527–0.604; *p* = 0.001, respectively).

## Discussion

This study offers a comprehensive evaluation of health disparities between tribal and non-tribal populations in India, particularly assessed socio-demographic, anthropometric, hemodynamic, hematological, blood glucose, lipid, hepatic, renal, and micronutrient variables. Moreover, our findings further addressed the association and predictive accuracy of clinical markers associated with cardiovascular burden to predict glycemic risk in tribal and non-tribal population.

The present study revealed marked socio-demographic and behavioral disparities between tribal and non-tribal populations. Tribal participants exhibited lower levels of educational attainment and a higher prevalence of tobacco and alcohol consumption, consistent with findings from previous studies including analyses from the NFHS-5 which reported that tribal have lower literacy, and school completion than non-tribal populations, reflecting structural barriers such as geographic isolation, poverty, and inadequate educational infrastructure (19). These educational inequities have also been attributed to socioeconomic disadvantage and limited institutional access (20). The elevated rates of smoking, tobacco chewing, and alcohol consumption among tribal groups align with earlier reports emphasizing the cultural acceptance of certain tobacco products, intergenerational transmission of habits, and the scarcity of targeted community intervention programs as key contributing factors (21). Recent analyses by Kishore et al. (22) identified several key predictors of hypertension, including male sex, advancing age, higher educational attainment, marital status, smoking, and alcohol use. Notably, their observation that higher educational attainment paradoxically increased hypertension risk among tribal contrary to typical epidemiological patterns. This unexpected pattern points to ongoing socioeconomic transitions within tribal communities (22, 23). This aligns with our observation of significant educational disparities between tribal and non-tribal groups, with 47.6% of tribal participants lacking formal education. At the same time, metabolic risk factors commonly linked to urbanization are becoming increasingly visible in these communities. The NFHS-5 findings also reinforce the urgency of addressing tobacco use. In our study, 30.3% of tribal participants reported current tobacco chewing, closely mirroring the national tribal prevalence of 28.5% (22). Together, these patterns point to a shifting risk profile that demands targeted and culturally responsive public health strategies.

Anthropometric differences were consistent across age and sex strata. Tribal groups exhibited lower BMI, weight, height, waist circumference, and hip circumference, aligning with NFHS-5 data and recent surveys that have highlighted persistent undernutrition and stunting in tribal populations (24, 25). These deficits likely reflect the combined effect of dietary patterns, nutritional transitions, and barriers such as poor education, lack of resources that stop improvement from one generation to the next. The hemodynamic profile observed in our study particularly the comparatively lower SBP and DBP levels among tribal participants differs from recent reports highlighting a growing hypertension burden in several tribal communities across India. For instance, Babu et al. (26), in a large multicentric study spanning five states, reported hypertension prevalence rates of 34.0% among tribal men and 28.3% among tribal women. Although nearly half (47.6%) of hypertensive individuals were aware of their diagnosis suggesting some improvement in health awareness treatment outcomes remained far from optimal. Notably, 60.1% of those receiving antihypertensive therapy had uncontrolled blood pressure, pointing to persistent gaps in long-term management and continuity of care (26). Similarly, Meshram et al. (27) documented an even higher hypertension prevalence (47.3%) among tribal adults in the Bastar district of Chhattisgarh, alongside striking rates of central obesity (67%) and tobacco use (78%) (27). These findings illustrate the marked regional heterogeneity in cardiovascular risk across tribal populations. Importantly, our observation that systolic hypertension independently predicted elevated HbA1c levels in the tribal cohort (*OR* = 5.530, *p* = 0.015) reinforces prior reports positioning hypertension as a key early marker of broader cardiovascular vulnerability in these communities.

Hemoglobin levels were lower among tribal men and women than non-tribal group, consistent with prior studies reported high anemia prevalence in tribal groups, which may be due to limited dietary diversity and a higher burden of infections such as malaria, hookworm, and other parasitic infestations common in tribal regions as documented in previous findings (28, 29). High RBC counts with low WBC and platelet counts were observed in tribal individuals, consistent with previous reports (30, 31), and may reflect the body's adaptation to moderate altitude environment and nutritional deficiency (32). Increased eosinophil counts further suggest ongoing parasitic exposure in forest and rural dwelling communities (29).

Lipid profiles showed lower total cholesterol, triglycerides, and LDL cholesterol, with higher HDL cholesterol in tribal men, resonant with earlier findings (33). Glycemic measures, including fasting glucose and HbA1c, were significantly lower in both sexes and across all age groups, supporting reports of lower diabetes prevalence among tribal populations, often attributed to lower adiposity and higher physical activity (34, 35). Slightly high random glucose levels in tribal men may reflect dietary variation and occupational physical exertion (36). Our observation that tribal participants had significantly lower fasting glucose and HbA1c levels is consistent with findings from recent multi-centric studies highlighting the uneven distribution of diabetes across tribal communities in India. Recently, Mallikarjuna et al. (37) reported an overall diabetes prevalence of 6.8% among tribal populations, with striking regional differences. Interestingly, their observation that waist circumference emerged as a significant independent predictor of diabetes among tribal participants rather than BMI alone reinforcing the need for population-specific anthropometric cutoffs and aligns closely with our findings stated that conventional obesity measures were not significantly associated with dysglycemia in the tribal cohort. Despite a high burden of modifiable risk factors (83.3% dyslipidemia and 27.9% hypertension), tribal populations in South India still showed a relatively low diabetes prevalence (2.4%) compared to neighboring non-tribal communities (37). This pattern hints at the protective influence of traditional lifestyles that may attenuate metabolic risk which mirrors our observation of favorable lipid profiles (lower total cholesterol, triglycerides, and LDL with higher HDL) coexisting with significant undernutrition and anemia in tribal participants. Together, these findings underscore the complexity of health transitions in tribal populations. Metabolic risk doesn't follow a single, uniform pathway; instead, it reflects diverse and context-specific trajectories shaped by geography, culture, and lifestyle. Collectively, these results underscore the coexistence of undernutrition with relative protection against metabolic disorders, as described in dual-burden analyses (38, 39).

Serum creatinine was lower among tribal participants, consistent with previous findings attributing this to lower body mass and muscle content rather than renal impairment (40). Liver enzyme profiles revealed high SGOT levels in both sexes and comparatively higher SGPT levels among tribal women. Similar trends have been observed in other tribal population-based studies (41), suggesting potential links to alcohol consumption, environmental and dietary exposures prevalent in some rural and tribal settings. These include hepatotoxic agents such as pesticide residues from agricultural practices, chronic exposure to toxic metals (arsenic, cadmium, lead), and dietary mycotoxins (aflatoxins), all of which are known to cause hepatocellular injury and elevate transaminase levels. The observed elevation in liver enzymes may also have a genetic basis, as variants in genes such as PNPLA3, TM6SF2, and MBOAT7 are known to influence hepatic fat accumulation and enzyme release, contributing to inter-population differences in SGPT and SGOT levels (42). The higher SGPT observed in women may also reflect sex-specific vulnerabilities, including poorer nutritional status, higher prevalence of untreated infections (such as hepatitis B in certain tribal communities), and the use of traditional or herbal remedies with hepatotoxic potential (42, 43).

Vitamin B12 levels were higher in tribal groups, possibly due to low excess to the RO processed drinking water in the tribal region, which is diverging from widespread deficiency reported in the general Indian population (44). Similar findings have been linked to traditional diets that include animal proteins and fermented foods (45). Conversely, folic acid levels were lower in younger adults, in line with prior reports of inadequate folate intake in rural Indian population (46). Vitamin D was marginally higher in younger adults, likely due to greater sun exposure, a trend supported by earlier studies (47).

Age-stratified analysis further revealed that younger tribal adults exhibited lower anthropometric indices, elevated eosinophil counts, and lower folic acid levels, indicating nutritional vulnerability in this age group findings consistent with those reported in previous studies (48). Middle-aged tribal populations often exhibit lower fasting glucose, HbA1c, and SBP and DBP levels than non-tribal groups, despite the high burden of anemia and leukopenia in tribal group, a pattern also reported in Indian tribal surveys (49). This phenomenon may reflect higher physical activity, traditional diets low in refined carbohydrates, and lower obesity rates that may collectively reduce metabolic risk. Furthermore, one study reported that anemia and hemoglobinopathies can shorten red blood cell lifespan, resulting in low HbA1c measurements (50). Chronic infections and undernutrition may also impair hematopoiesis and glucose metabolism, contributing to lower glycemia despite compromised overall health. In our study, the older tribal population exhibited comparatively favorable glycemic and lipid profiles, consistent with previous reports (51). This pattern may be explained by continued engagement in physically demanding activities, adherence to traditional diets rich in unprocessed foods, and lower adiposity, all of which collectively reduce the risk of metabolic disorders (48). Comparisons across regions highlight the considerable geographic variation in aging patterns and metabolic health among tribal communities. In a recent study from Northeast India, Mounika and colleagues (18) examined cardiovascular risk among tribal populations and reported markedly different risk profiles between the two states. In Assam, smoking prevalence was particularly high (66.2%), while dietary practices varied substantially due to geographic isolation. Using WHO/ISH cardiovascular risk prediction charts, the authors found that tribal population of Assam and Mizoram were at high risk for cardiovascular diseases, with the burden concentrated largely among older (18). This age-related gradient closely mirrors our findings, which demonstrate a steady rise in glycemic risk with advancing age. Notably, the Northeast study identified age and SBP as the most influential determinants of cardiovascular risk. This observation is consistent with our ROC analysis, where both SBP (*AUC* = 0.725) and age (*AUC* = 0.687) showed acceptable discriminatory performance in predicting glycemic risk among tribal participants. Taken together, these findings spanning geographically distinct tribal regions from the Western Ghats to the Northeastern hills and Central India underscore the value of simple, age and blood pressure based screening approaches. Such strategies may offer practical and scalable options for early cardiovascular risk detection in resource-constrained tribal settings.

Regression and ROC analysis revealed age as the strongest predictor of glycaemic progression in both tribal and non-tribal populations, with non-tribal participants demonstrating relatively higher odds ratios for overt diabetic HbA1c level. Obesity and hypertriglyceridemia exhibited stronger associations within the non-tribal group, whereas elevated SBP emerged as a key correlate of dysglycaemia among tribal participants. Distinct population-specific lipid patterns were also observed, including an inverse association between HDL cholesterol and prediabetic HbA1c level in the tribal cohort. ROC curve analyses demonstrated modest-to-moderate discriminatory performance for most evaluated biomarkers, with higher predictive accuracy for overt diabetic HbA1c level compared to prediabetic HbA1c level. These observations are consistent with findings from large-scale Indian epidemiological studies, including the Longitudinal Ageing Study in India and the ICMR-INDIAB Study, which have demonstrated that advancing age independently predicts impaired glucose tolerance and type 2 diabetes, even after adjustment for adiposity and socioeconomic determinants. Such evidence reinforces the central role of biological aging in glycaemic deterioration across diverse Indian populations (1, 35). Furthermore, the consistent effect of age across both populations is biologically plausible and aligns with established mechanisms underlying β-cell senescence, mitochondrial dysfunction, and progressive insulin resistance (52). Advancing age is associated with reduced β-cell compensatory capacity, increased oxidative stress, and chronic low-grade inflammation, collectively shifting glucose homeostasis toward hyperglycaemia. The higher odds of overt diabetic HbA1c level observed among older non-tribal individuals may reflect prolonged exposure to obesogenic, sedentary behavior, and refined carbohydrate intake, accelerating age-related metabolic deterioration (53).

Systolic hypertension emerged as a prominent predictor of dysglycaemia within the tribal cohort, whereas prehypertension was associated with elevated HbA1c levels among non-tribal participants. These observations align with the well-established bidirectional relationship between insulin resistance and hypertension, mediated through endothelial dysfunction, sympathetic nervous system over activity, activation of the renin-angiotensin-aldosterone system (RAAS), and chronic low-grade inflammation (54). Notably, among tribal participants, SBP demonstrated stronger discriminatory performance for overt diabetic HbA1c level than age (AUC = 0.725), suggesting that vascular alterations may function as an early integrative marker of metabolic stress in populations with relatively low obesity prevalence. Comparable findings have been reported in rural Indian cohorts, where elevated SBP independently predicted incident diabetes (55). Collectively, these results support the implementation of glycaemic screening in hypertensive tribal adults, even in the absence of overt obesity, to facilitate earlier identification and intervention.

Adiposity-related indicators, including overweight and obesity, were significantly associated with prediabetic and diabetic HbA1c levels among non-tribal participants, whereas similar associations were not observed in the tribal cohort. This divergence likely reflects differences in body composition, regional fat distribution, and lifestyle exposures between the two populations. In urban and semi-urban Indian settings, visceral adiposity is strongly linked to insulin resistance through mechanisms involving adipokine imbalance characterized by reduced adiponectin and elevated leptin and TNF-α along with ectopic lipid deposition and increased hepatic gluconeogenesis (56). By contrast, tribal populations, who generally exhibit lower BMI and higher levels of habitual physical activity, may demonstrate a distinct metabolic phenotype in which dysglycaemia is less tightly coupled to overall adiposity (57). These findings support the concept of heterogeneous health architectures across communities and underscore the need for caution in applying uniform BMI-based screening thresholds without accounting for population-specific metabolic contexts.

Triglycerides and LDL cholesterol emerged as significant predictors of dysglycaemia among non-tribal participants, with triglycerides demonstrating moderate discriminatory performance for overt diabetic HbA1c level (*AUC* = 0.689). Elevated triglyceride concentrations reflect hepatic insulin resistance and increased very-low-density lipoprotein (VLDL) production central features of the atherogenic dyslipidaemia frequently observed in type 2 diabetes (58). Beyond serving as metabolic markers, high triglyceride levels are increasingly recognized as contributors to insulin resistance through lipotoxic effects and impairment of intracellular insulin signaling pathways (59). The observed association between LDL cholesterol and prediabetic HbA1c level further underscores the clustering of cardiovascular abnormalities in populations undergoing nutritional and epidemiological transition, consistent with findings from Indian cohort studies (60). Collectively, these results suggest for integrated lipid-glucose screening strategies in non-tribal and urbanizing communities to enable early detection and comprehensive cardiovascular risk management.

Interestingly, HDL cholesterol levels demonstrated an inverse association with prediabetic HbA1c among tribal participants. Although this finding appears counterintuitive, it may reflect residual confounding related to nutritional status or inflammatory burden. HDL concentrations can be influenced by chronic infections, parasitic infestations, and protein-energy undernutrition, all of which remain prevalent in certain vulnerable populations (61). Moreover, in communities with relatively low overall cardiovascular burden, variability in HDL levels may not correspond to insulin resistance in the same manner observed in obesity-driven metabolic syndrome (62). These observations highlight the importance of interpreting lipid-glycaemic relationships within the appropriate nutritional and epidemiological context, particularly in populations experiencing dual burdens of undernutrition and emerging metabolic risk. Sex was not independently associated with glycaemic categories in either population after multivariable adjustment. This finding is consistent with evidence from Indian national surveys demonstrating that apparent sex differences in diabetes prevalence are substantially attenuated after controlling for age, adiposity, and behavioral determinants (63). Furthermore, the study supports the development of population-tailored public health strategies that are aligned with ongoing national genomic efforts, such as the Genome India Initiative, thereby advancing precision public health approaches in India.

## Limitations

The cross-sectional design limits the capacity to establish temporal relationships between clinical indicators and glycemic status i.e., whether changes in clinical indicators occurred before or after changes in blood sugar levels. Although we analyzed the data separately by age and sex, other important factors such as diet, physical activity, detailed socioeconomic conditions, and environmental exposures were not fully measured and may have influenced the results. Information on behavioral variables was self-reported and may therefore be subject to social desirability bias. Biomarkers were measured only once, so they may not represent long-term metabolic patterns or natural variations over time. The participants were selected from specific districts in two states, which may limit how well the findings apply to other tribal and non-tribal populations across India. Differences within individual tribal groups could not be examined due to confidentiality requirements. Overall, these limitations should be considered when interpreting the strength and wider public health relevance of the findings.

## Conclusion

This cross-sectional study provides a comprehensive assessment of health disparities between tribal and non-tribal populations in western and central India. The findings demonstrate a clear epidemiological and metabolic divergence between these communities. Tribal populations continue to experience a dual burden of disease, characterized by persistent undernutrition, anemia, hematological alterations, and micronutrient deficiencies alongside emerging cardiovascular risk patterns. Although tribal participants exhibited lower anthropometric indices, hemoglobin concentrations, and leukocyte and platelet counts, they displayed comparatively more favorable lipid and glycemic profiles across age and sex strata. Notably, advancing age and elevated SBP were significant predictors of glycemic progression within tribal groups, suggesting an early transition toward metabolic risk. In contrast, non-tribal populations showed stronger associations between adiposity, dyslipidemia, and impaired glycemic regulation. The differential predictive performance of conventional cardiovascular indicators underscores the importance of developing population-specific risk assessment models and integrated prevention strategies to address evolving health disparities.

## Data Availability

The raw data supporting the conclusions of this article will be made available by the authors, without undue reservation.
